# 28-Year Survival following Several Metastasectomies, Going through 8th Line Systemic Therapy in a Case of mRCC

**DOI:** 10.1155/2015/523258

**Published:** 2015-02-25

**Authors:** A. Magdy, K. Bretterbauer, S. Hruby, T. Kunit, D. Colleselli, G. Janetschek, M. Mitterberger

**Affiliations:** Universitätsklinikum Salzburger Landeskrankenhaus (SALK), 5020 Salzburg, Austria

## Abstract

Metastatic renal cell carcinoma (mRCC) has been one of the most treatment-resistant cancers because of its unpredictable clinical course, resistance to chemo- and radiotherapy, and the limited response to immunotherapy and targeted agents. We present a case of long-term survival, that is, 28 years, after primary diagnosis (longest survival in the literature up to our knowledge) with mRCC after several metastasectomies (from local site recurrence, liver, and lung) and eight lines of systemic targeted therapy. This case report shows how crucial is the regular follow-up of patients with RCC after primary management and positive impact of early metastasectomy and systemic targeted therapy in case of mRCC on patients' condition and overall survival.

## 1. Case Report

The patient was 50 years old (1987) when he suffered from a 4 cm left renal mass with no evidence of metastasis for which left radical nephrectomy was performed. Histopathology revealed clear-cell renal cell carcinoma (cRCC), grade II, stage pT1a, with negative surgical margins. The patient was then followed up regularly.

In 1995, the patient had a malignant melanoma excised from the left arm. He also underwent Whipple operation in 1996 for a neuroendocrine tumor of the pancreatic head that recurred in February 2000 and partial resection of the pancreas with intraoperative irradiation of surgical bed was done.

In October 2000, lower cuts of the contrast-enhanced chest CT revealed a small 8 mm basolateral left pulmonary nodule and metastasectomy was performed. Histopathology revealed the first reported recurrence of the cRCC 13 years after the initial diagnosis with negative surgical margins.

In November 2001, the patient underwent right lobar thyroidectomy for asymmetrical thyroid enlargement revealing metastasis of the cRCC with negative surgical margins. Afterwards the patient started interferon-alpha (INF-*α*), interleukin-2 (IL-2), and 5-fluoruracil for 8 weeks as first- line adjuvant systemic therapy for mRCC.

**Figure 1:**
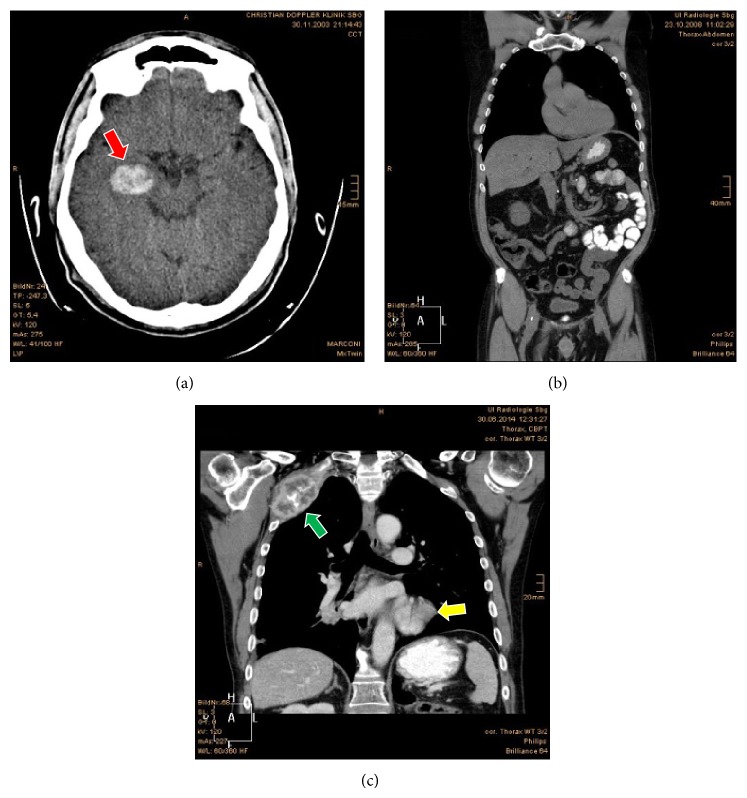
(a) Red arrow: brain CT axial view showing brain metastasis at the right temporal lobe in 2003. (b) Thorax, abdomen, and pelvis CT coronal view showing no evidence of metastasis after multiple metastasectomies. The scan was performed in 2008. (c) Recent (2014) chest CT coronal view showing a metastatic focus on the left lower lung lobe (yellow arrow) as well as an osteoclastic lesion involving the upper two ribs (green arrow).

In December 2003, brain CT for severe headache was performed, revealing a 3 × 2 cm focal metastasis in the brain, likely of cRCC type, with temporal lobe hemorrhage. The patient survived the incident and received palliative radiotherapy on this lesion in 2003, 2004, and 2005 (see Figure 1).

In November 2007, 20 years after the first renal tumor therapy, a 5 cm local tumor recurrence appeared at the left retroperitoneum in the contrast-enhanced CT scan. The cancer recurrence was surgically excised. Recurrence was also observed in the remaining part of the thyroid gland (4 cm), which was again excised and revealed cRCC. Therefore, a second line of systemic therapy with sunitinib (Sutent 50 mg once daily, 4 weeks on and 2 weeks off) was started. The patient received 30 cycles of sunitinib until September 2011.

In September 2011, a suspicious 3 cm hepatic focal lesion appeared in the regular follow-up which was operated on through laparotomy and excision of segment IVa, revealing cRCC, GII. Therefore, the regimen was shifted in November 2011 to a third line of systemic therapy, 10 cycles of temsirolimus (Torisel 25 mg, Pfizer; i.v. injection over 30–60 minutes) till follow-up CT in January 2012 revealed small subpleural focal lesion (2 mm) and a posterobasal lesion in the lower left lung lobe (1 cm). The regimen was shifted to a fourth line of systemic therapy with oral sorafenib twice daily (Nexavar 400 mg, Bayer).

In October 2012, the follow-up CT showed progressive enlargement of the pulmonary metastasis up to 4 cm and appearance of a new focus at the apical part of the lower right lung lobe and five metastatic foci in the liver. The institutional tumor board decided to start with the fifth line of therapy, bevacizumab (Avastin, Genentech Roche) 10 mg/kg i.v. injection every 2 weeks and INF-*α* 3 *μ*IU subcutaneous injection three times/week since January 2013. The patient received 18 consecutive cycles of this therapy.

In November 2013, two osteolytic bone lesions appeared in two right ribs and the lung metastases progressed. The local tumor board decided radiotherapy of the osteolytic lesions and to start the sixth line therapy with axitinib (Inlyta, P fizer; 5 mg twice a day). Due to intolerable side effects (fatigue and diarrhea), the patient soon stopped the medication on his own.

In December 2013, a seventh line therapy was initialized with everolimus (Afinitor, Novartis; 10 mg once daily). Because of progressive enlargement of the lung, liver, and right hilar LN metastases, treatment shifted in June 2014 to eighth line of systemic therapy for mRCC, pazopanib (Vortrient, GSK, 400 mg oral tablets twice daily).

**Figure 2:**
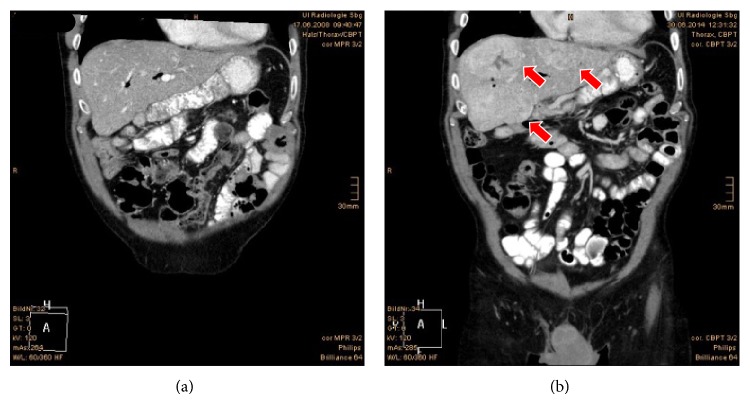
(a) Abdomen CT coronal view showing no evidence of metastasis after multiple metastasectomies. The scan was performed in 2008. (b) A recent (2014) picture of the same view showing multiple liver metastases (red arrows) before starting the 8th systemic targeted therapy.

Currently, the patient is still coming for regular follow-up checks to our clinic, getting the pazopanib (his 8th line of systemic treatment) as scheduled up to the day. However, the patient is suffering from multiple metastatic symptoms in liver, ribs, and lung as previously described (see Figure 2).

## 2. Discussion

Management of RCC has evolved over the last several decades as imaging and therapies have responded to the challenges of diagnosis and treatment [1]. Currently, more than 50% of RCCs are detected incidentally when noninvasive imaging is used to investigate a variety of nonspecific symptoms and other abdominal diseases [2]. Oncologic control is mostly favorable for localized RCC, as surgical excision results in cure. Unfortunately, between 20% and 30% of patients with RCC will present with metastatic disease at the initial diagnosis, and as many as 40% will demonstrate metastasis after primary surgical treatment for localized RCC [1].

mRCC has always been considered a therapeutic challenge for clinicians due to its chemo- and radioresistance, as well as its limited response to immunotherapy and targeted therapy [2, 3]. The median time before a relapse after nephrectomy is 15 months, and 85% of relapses occur within 3 years [4].

In our patient, tumor relapse occurred 13 years after primary management of the RCC. Metastasis can occur in unusual sites, including the thyroid gland, pancreas, skeletal muscle, and skin or underlying soft tissue [5]. The role of metastasectomy for the treatment of metastasis from RCC is widely accepted. In carefully selected patients, progression-free survival (PFS) and disease-specific survival (DSS) improve with metastasectomy compared to patients who do not undergo multimodal therapy or after an incomplete resection [1].

This case report shows that, with multiple complete metastasectomies, the overall survival can be extended.

Only a minority of patients treated with immunotherapy for metastatic RCC (mRCC) exhibit an objective response, and complete remission is rare [6]. Recent advances in molec ular biology have led to the development of several novel drugs for mRCC. The introduction of seven new agents in the past 8 years has transformed the systemic treatment of mRCC, improving prognosis from a median overall survival (OS) of approximately 1 year to >2 years [1]. The new agents include four multitargeted tyrosine kinase inhibitors (TKIs), sorafenib [2], sunitinib, pazopanib [3], and axitinib [4]; the humanized antivascular endothelial growth factor (VEGF) monoclonal antibody bevacizumab with interferon- (IFN-) *α*2a [5]; and two mammalian target of rapamycin (mTOR) complex 1 kinase inhibitors, temsirolimus [6], and everolimus [7]. Compared to immunotherapies, the objective response of targeted agents in the primary tumor and its metastatic lesions correlate with improvements in PFS and OS [6].

The patient in this case report was treated with all available lines of targeted agents available at the time for mRCC. Despite the fact that the patient never participated in a study, he is still a long-term survivor of mRCC.

## 3. Conclusion

Despite multiple morbidities and treatment-related side effects, long-term survival can be reached in cases of mRCC with multiple complete metastasectomies. The use of systemic targeted therapies may render mRCC a chronic disease, rather than a rapid lethal disease.
